# Supplementary material for: Characterization of CrufCSP1 and Its Potential Involvement in Host Location by Cotesia ruficrus (Hymenoptera: Braconidae), an Indigenous Parasitoid of Spodoptera frugiperda (Lepidoptera: Noctuidae) in China
Source: Insects. 2023 Dec 1;14(12):920. doi: 10.3390/insects14120920 (PMC10744196; doi:10.3390/insects14120920)
Supplement: Supplementary file 1 [file insects-14-00920-s001.zip › insects-2685742-supplementary.pdf]

Supplementary Materials:

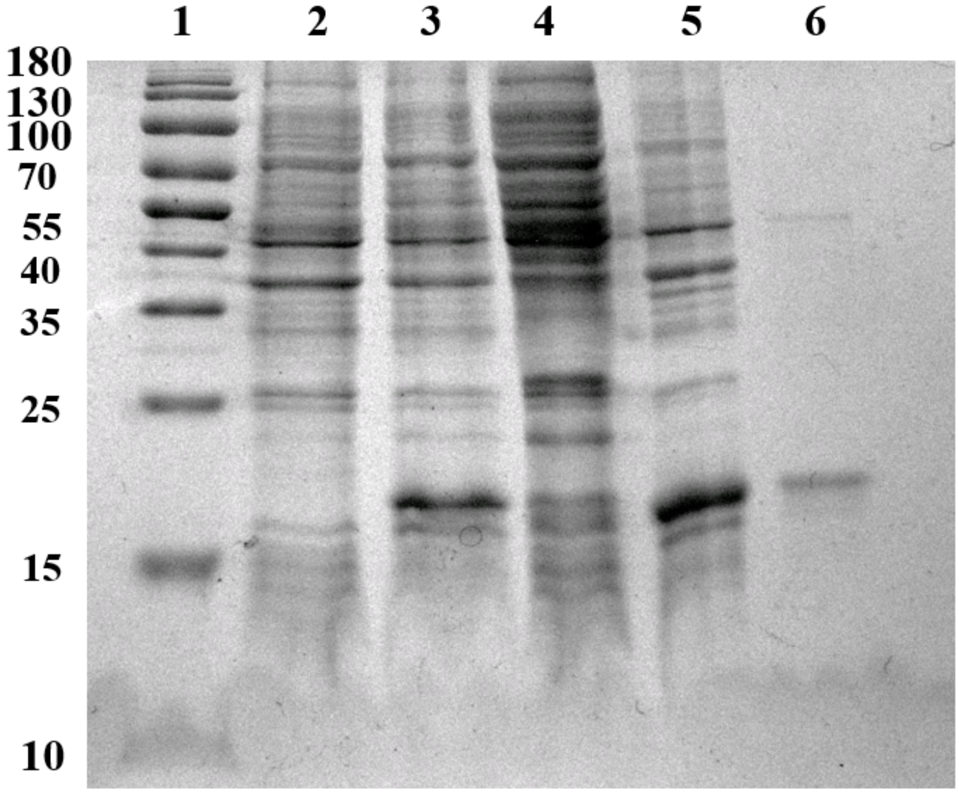

Figure S1. SDS-PAGE analysis for the expression and purification of CrufCSP1. M, protein marker; lane 1, non-induced PET30a/CrufCSP1 before IPTG induction; lane 2, IPTG-induced PET30a/CrufCSP1; lane 3, supernatant of induced PET30a/CrufCSP1; lane 4, sediment of induced PET30a/CrufCSP1; lane 5, purified CrufCSP1 with His-tag.

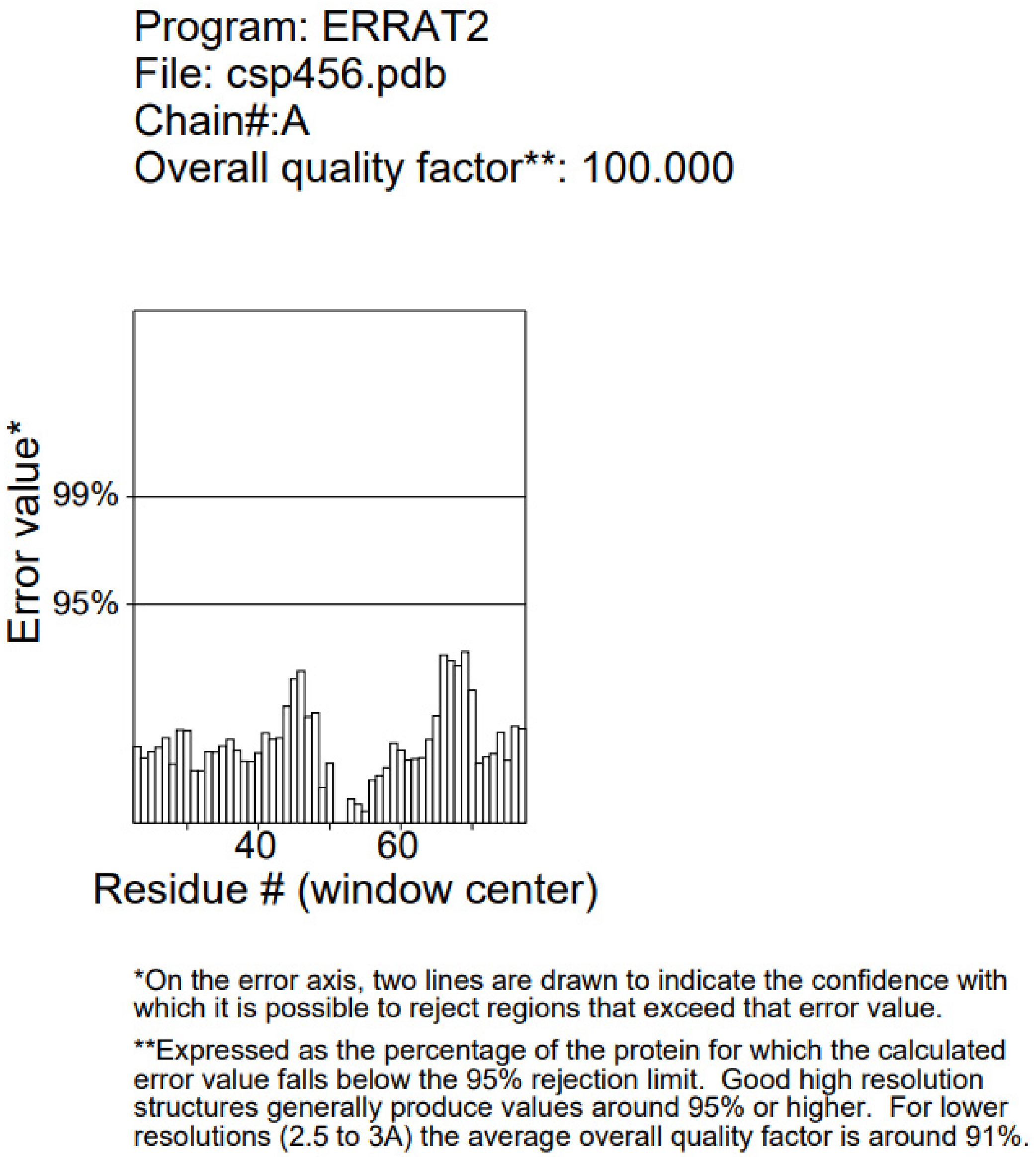

Figure S2. ERRAT results of the constructed 3D model of CrufCSP1.

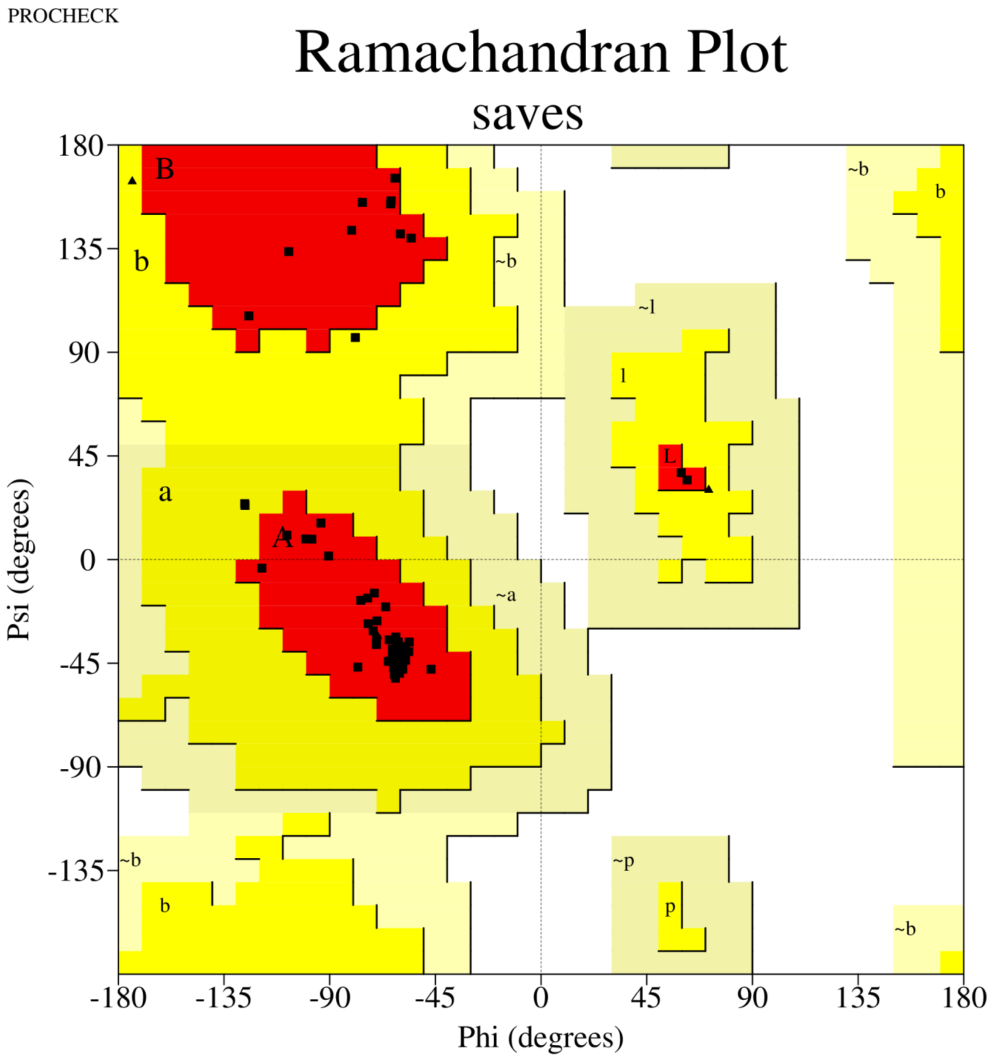

Plot statistics

Residues in most favoured regions [A,B,L]	52	94.5%
Residues in additional allowed regions [a,b,l,p]	3	5.5%
Residues in generously allowed regions [~a,~b,~l,~p]	0	0.0%
Residues in disallowed regions	0	0.0%
	----	------
Number of non-glycine and non-proline residues	55	100.0%
Number of end-residues (excl. Gly and Pro)	2	
Number of glycine residues (shown as triangles)	4	
Number of proline residues	2	

Total number of residues	63	

Based on an analysis of 118 structures of resolution of at least 2.0 Angstroms and R-factor no greater than 20%, a good quality model would be expected to have over 90% in the most favoured regions.

Figure S3. Ramanchandran plot of the constructed 3D model of CrufCSP1.
